# Mercury in Different Feather Types from Great Cormorants (*Phalacrocorax carbo* L.) Inhabiting the Vistula Lagoon Ecosystem in Poland

**DOI:** 10.1007/s00128-012-0771-z

**Published:** 2012-08-24

**Authors:** Małgorzata Misztal-Szkudlińska, Piotr Szefer, Piotr Konieczka, Jacek Namieśnik

**Affiliations:** 1 Department of Food Sciences, Medical University of Gdansk, al. Gen. J. Hallera 107, 80-416 Gdańsk, Poland; 2 Department of Analytical Chemistry, Chemical Faculty, Gdansk University of Technology, ul. Narutowicza 11/12, 80-952 Gdańsk, Poland

**Keywords:** Mercury, Feathers, Cormorant, CV-AAS

## Abstract

Total mercury levels in different feather types (down, contour, tail and flight) in Great Cormorants (*Phalacrocorax carbo* L.) were determined using CV-AAS. Feathers from Great Cormorants inhabiting the Vistula Lagoon ecosystem have an average Hg level of 7.14 ± 3.99 (μg/g w.w.). Age-dependent concentrations of Hg were statistically significant (ANOVA Kruskal–Wallis, *p* < 0.0001). There were also significant differences in Hg levels in different parts of feathers from adults and immature birds (ANOVA Kruskal–Wallis, *p* < 0.0001). Cormorant chick feathers appear to be a potential biomonitor of Hg pollution in the Vistula Lagoon ecosystem, but this subject requires further research.

In aquatic environments Hg is converted to methylmercury and in this form is rapidly incorporated into the food chain. Aquatic birds, which are top predators in the food chain, may be exposed to significant concentrations of Hg. Its toxic effects in birds include reduced food intake, leading to weight loss; progressive weakness in the wings and legs, making flight, walking and standing difficult; an inability to coordinate muscle movements. High Hg levels in birds most often affect their immune, detoxification and nervous systems; they also impair reproduction (Boening 2000). On the basis of a limited number of data, Burger and Gochfeld (2000) claimed that feather Hg levels from 5 to 40 μg/g d.w. led to lower reproduction and survival. Feathers of fish-eating birds could serve as good monitors of spatial and temporal patterns of Hg contamination in water ecosystems (Ochoa-acuña et al. 2002).

Metal pollutants can be incorporated into birds’ feathers along three routes: from the blood during feather growth, from the excretion of salt or the secretion of preen glands, and through contact with the habitat (Goede and de Bruin 1984). Feathers may serve as a useful indicator of inorganic pollutants because concentrations of metals correlate well with their internal levels during the time of feather formation. Moreover, mercury levels in feathers are stable, and the metal may bind to the sulphydryl groups of the keratin as the feathers grow. The most important pathway of mercury elimination in birds is its “excretion” when the feathers are moulted (Ochoa-acuña et al. 2002; Dauwe et al. 2003). The aim of the present study was to analyse total mercury levels in feathers from Great Cormorants inhabiting the Vistula Lagoon and to compare them with concentrations reported from the feathers of other aquatic birds.

## Materials and Methods

Different parts of feathers were taken from 62 Great Cormorants. These birds are present in Poland during the breeding season from February or March to September. In this country they enjoy partial species protection status (Dz.U. z 2004 r. Nr 220, poz. 2237). The breeding colony (a nature reserve) from which feather samples were taken is situated at Kąty Rybackie (54°21′N, 19°14′E) near the Vistula Lagoon; this is the largest breeding colony of the Great Cormorant in Europe.

The cormorants were captured in 2006 by permission of the local environment protection authorities and were aged on the basis of plumage characteristics. The feathers of adult cormorants are black with white spots on the cheeks and thighs. Immature birds have a white belly with a variable number of dark spots. There is no sexual dimorphism. The moult strategy in cormorant is difficult to interpret because birds replace feathers singly during the course of the season (Nelson 2005).

**Fig. 1:**
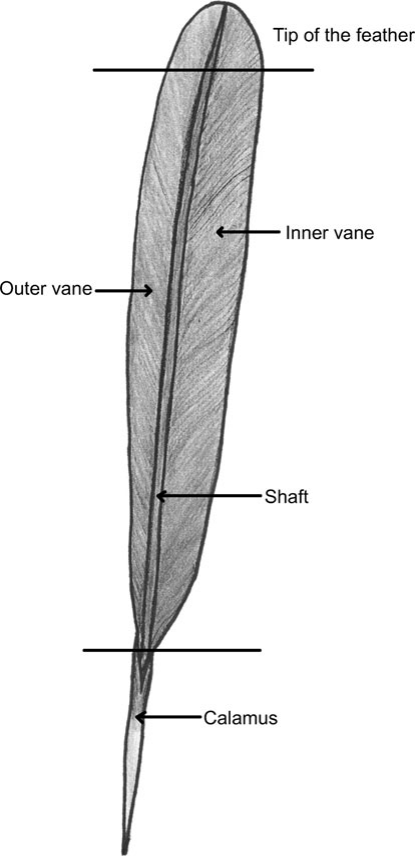
Feather parts

The birds were segregated as follows: 7 chicks, 11 immatures (5 females, 6 males) and 44 mature birds (20 females, 24 males). Down, contour, flight and tail feathers were taken from adult and immature specimens, but only the tail feathers from the chicks. The tail feathers from 18 adult and immature birds were cut up into the feather tip, inner vane, outer vane, shaft and calamus (Fig. 1).

The feather samples were rinsed three times—with water + detergent, then with distilled water + acetone (1:1) and finally with Milli-Q water—after which they were dried overnight at room temperature to constant mass (Saeki et al. 2000). The samples were homogenized and then decomposed at 800°C in a flow of clean air. Hg was determined by CV-AAS at 253.65 nm (MA-2000 Mercury Analyzer). Three analytical subsamples were prepared from each sample. A pooled sample consisted of 3–5 feathers. Quality was assured by analyses of certified reference materials, i.e. CRMs: DORM-2 (National Research Council, Canada) and BCR-463 (IRMM, Belgium). Recoveries of total Hg were 101.0% and 97.1% respectively and the SDs were 0.09% and 0.09% respectively. The limit of detection for the method was 0.096 ng and the limit of quantification was 0.29 ng. Other validation parameters for the analytical method were reported by Konieczka et al. (2010).

The statistical analyses were performed using the STATISTICA 9.0 for Windows (Copyright^©^ StatSoft, Inc. 1984–2010) and Graph Pad Prism 5.0. The data were processed using the ANOVA Kruskal–Wallis test to check for any significant (*p* < 0.0001) difference between Hg concentration and age. Where a statistically significant variation was found, the post hoc Dunn test was applied to seek a more detailed relation. Dunn’s post hoc test compares the difference in the sum of ranks between two columns with the expected average difference (Motulsky 2005).

## Results and Discussion

The average total Hg concentration in cormorant feathers was 7.14 ± 3.99 μg/g d.w. (0.63–1.20 μg/g d.w.). The average Hg level in chick feathers was 1.16 ± 0.5 μg/g d.w. (Table 1).

**Table 1:** Mercury levels in Great Cormorant feather types

Feather type	Great Cormorant (*Phalacrocorax carbo*) Hg ± SD and range (μg/g w.w.)				
	Adult (N = 44)		Immature (N = 11)		Together (N = 55)
	Female (N = 20)	Male (N = 24)	Female (N = 5)	Male (N = 6)	
Down	9.26 ± 3.92 (3.57–16.7)	9.45 ± 3.59 (3.63–18.0)	7.75 ± 1.05 (6.95–9.28)	7.52 ± 4.64 (1.97–12.3)	8.89 ± 3.75 (1.97–18.0)
Contour	10.6 ± 6.69 (2.5–27.4)	8.98 ± 4.55 (2.54–21.8)	7.27 ± 1.29 (6.12–9.09)	8.47 ± 8.1 (2.09–24.2)	9.22 ± 5.71 (2.09–27.4)
Tail	5.57 ± 4.2 (1.57–17.1)	7.01 ± 4.22 (2.85–20.0)	4.72 ± 4.8 (1.69–10.3)	8.83 ± 8.99 (1.59–28.1)	6.68 ± 5.14 (1.57–28.1)
Flight	6.08 ± 3.42 (1.72–13.9)	6.89 ± 4.56 (1.65–19.4)	2.16 ± 1.19 (1.33–3.91)	7.52 ± 9.56 (1.38–28.5)	6.32 ± 4.99 (1.33–28.5)
Total	8.18 ± 4.2 (1.72–19.4)	7.66 ± 3.03 (2.67–17.3)	5.84 ± 1.66 (3.49–7.2)	9.27 ± 4.14 (4.77–17.4)	7.92 ± 3.56 (1.72–19.4)

**Fig. 2:**
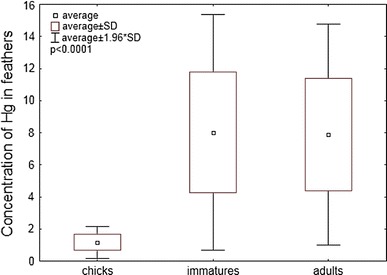
Concentrations of the total Hg (μg/g d.w.) in all the feathers of chicks, immatures and adults of Great Cormorants inhabiting the Vistula Lagoon ecosystem

Figure 2 shows that there are statistically significant age-dependent variations in Hg concentrations in feathers (ANOVA Kruskal–Wallis, H = 18.14, *p* < 0.0001). The post hoc Dunn test was used to check for a more specific relation. The concentration differences were significant in chicks and adults (test post hoc Dunn, *p* < 0.001), as well as in chicks and immature birds (test post hoc Dunn, *p* < 0.01).

**Fig. 3:**
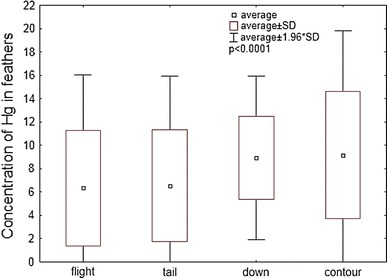
Concentrations of the total Hg (μg/g d.w.) in different types of Great Cormorant feathers from the Vistula Lagoon ecosystem

Figure 3 shows that there are also statistically significant variations in the Hg content of the different feather types from adults and immature birds (ANOVA Kruskal–Wallis, H = 30.37, *p* < 0.0001). The Dunn post hoc test revealed a statistically significant differentiation of concentration between contour tail feathers, contour and flights (*p* < 0.05), down tail feathers as well as down flights (*p* < 0.001).

There were no significant differences in Hg levels between the sexes.

Feathers of Great Cormorants from the Vistula Lagoon ecosystem contain significantly higher levels of Hg than their counterparts from urban areas in Tokyo, Lake Biwa and the Mie colony in Japan. According to Saeki et al. (2000) and Nam et al. (2005), Great Cormorant feathers contained ca. 3 μg Hg/g d.w. The mercury content in the Great Cormorant feathers in the present study was similar to that in feathers of *P. carbo* from the Caspian Sea in Iran (Rajaei et al. 2011): 7.92 ± 3.56 and 8 ± 1 μg/g d.w. respectively. Mazloomi et al. (2008) reported a mean level of 4.44 μg/g in cormorant feathers from the Fereidoonkenar region (Iran) in the southern Caspian Sea. *Phalacrocorax auritus* from two reservoirs in New Mexico had 4.01 and 2.34 μg Hg/g w.w. in the tail feathers (Caldwell et al. 1999). Very low levels of Hg (0.251 μg/g d.w.) were reported by Burger and Gochfeld (2001) in the feathers of Cape Cormorants (*Phalacrocorax capensis*) from Namibia (southern Africa). The feathers of the Great Cormorants that we analysed contained a concentration of Hg similar to that in Royal Albatrosses (6.8 μg/g d.w.) from the southern Indian Ocean and Laysan Albatrosses (7.2 μg/g d.w.) from the North Pacific, as well as Herring Gulls (6.06 μg/g d.w.) and Glaucous Gulls (5.96 μg/g d.w.) from Chaun in Siberia (Kim et al. 1996a, b). Boening (2000) and Ochoa-acuña et al. (2002) found some differences in feather Hg concentrations across taxonomic bird groups. They assumed that Hg contents in feathers depended on feeding strategies and to a lesser extent on differences in the metabolism and excretion of this metal. Mercury concentrations in birds also depend on body size, moult strategy, migration patterns and physiology (Stewart et al. 1997). Fish-eating birds like cormorants are at risk of higher contents of Hg because its circulation is associated mainly with water basins.

The results of this study show that Hg levels in feathers were lower in chicks than in adults. A similar dependence was reported by Stewart et al. (1997) in Kittwakes, Arctic Skuas and Common Skuas.

Examination of feather parts revealed only slight differences in Hg concentrations (μg/g d.w.) outer vane 7.38 ± 6.08, inner vane 7.83 ± 6.62, tip 7.32 ± 6.17, shaft 7.49 ± 5.7 and calamus 6.83 ± 5.7. This stands in agreement with the data given by Dauwe et al. (2003) and implies that mercury is evenly bound during feather formation.

Birds feathers are used as indicators of environmental pollution because the Hg content in the feathers reflects its content in the blood at the time of feather formation. Moreover, Hg levels in feathers are stable, feathers are easy to collect and their collection is non-invasive (Goede and de Bruin 1984; Burger and Gochfeld 2000; Boening 2000). Levels of mercury in growing feathers are directly and linearly related to its dietary intake by chicks of the same species of birds (Weech et al. 2006). It appears that cormorant chick feathers could be used as monitoring material for measuring the exposure of birds to Hg in the Vistula Lagoon ecosystem because the chicks are the most highly exposed to regional metal pollution. This suggestion requires further research, however.
